# Systematic Analysis of Lysine Lactylation in the Plant Fungal Pathogen *Botrytis cinerea*

**DOI:** 10.3389/fmicb.2020.594743

**Published:** 2020-10-26

**Authors:** Mingming Gao, Ning Zhang, Wenxing Liang

**Affiliations:** Key Lab of Integrated Crop Pest Management of Shandong, College of Plant Health and Medicine, Qingdao Agricultural University, Qingdao, China

**Keywords:** lysine lactylation, *Botrytis cinerea*, fungal pathogenicity, ribosomal protein, post-translational modification

## Abstract

Lysine lactylation (Kla) is a newly discovered histone post-translational modification (PTM), playing important roles in regulating transcription in macrophages. However, the extent of this PTM in non-histone proteins remains unknown. Here, we report the first proteomic survey of this modification in *Botrytis cinerea*, a destructive necrotrophic fungal pathogen distributed worldwide. After a global lysine lactylome analysis using LC-MS/MS, we identified 273 Kla sites in 166 proteins, of which contained in 4 types of modification motifs. Our results show that the majority of lactylated proteins were distributed in nucleus (36%), mitochondria (27%), and cytoplasm (25%). The identified proteins were found to be involved in diverse cellular processes. Most strikingly, Kla was found in 43 structural constituent proteins of ribosome, indicating an impact of Kla in protein synthesis. Moreover, 12 lactylated proteins participated in fungal pathogenicity, suggesting a potential role for Kla in this process. Protein interaction network analysis suggested that a mass of protein interactions are regulated by lactylation. The combined data sets represent the first report of the lactylome of *B. cinerea* and provide a good foundation for further explorations of Kla in plant fungal pathogens.

## Introduction

Post-translational modifications (PTMs) represent a crucial means of regulating diverse biological processes and cellular physiology by influencing protein structure and function. More than 300 types of PTMs have been described in recent reports, such as ubiquitination, glycosylation, methylation, phosphorylation, and acylation (Walsh et al., 2005; Witze et al., 2007). The application of liquid chromatography-mass spectrometry (LC-MS/MS)-based proteomics has been responsible for the discovery of many diverse protein modifications. The majority of novel histone PTMs are classified as short-chain Lys acylations. These modifications are similar to Lys acetylation (Kac), a well-studied modification of Lys on the ε-amine group, yet they are distinct in hydrocarbon chain length, hydrophobicity or charge (Sabari et al., 2017). Lysine acetylation was first reported on histones in the 1960s (Vidali et al., 1968). For a long time after, acetylation was considered as a major modification on histones regulating chromatin structure and gene transcription in cells. However, in recent years, there is a growing body of evidence demonstrating that acetylation also occurred in a diverse range of non-histone proteins (Glozak et al., 2005; Spange et al., 2009). Large numbers of researches of functions of acetylation proved that this modification was biologically significant, involved in regulation of diverse protein properties including subcellular localization, protein stability, transcription activity, and DNA-protein interactions (Narita et al., 2019). Other types of lysine acylation have been described in recent decades, such as crotonylation, succinylation, butyrylation, malonylation, propionylation, 2-hydroxyisobutyrylation, and glutarylation in histones and other types of proteins (Chen et al., 2007; Tan et al., 2011; Dai et al., 2014; Hirschey and Zhao, 2015). Thus, global proteomic analyses have broadened our understanding of lysine acylation and suggested that it affects a wide range of biological functions.

Histone lysine lactylation (Kla) is a newly discovered histone modification, which regulates gene expression in macrophages. In M1 macrophages, lactate is derived from incompletely oxidized glucose and then generate lactyl-CoA, which is transferred to lysine tails of histone proteins via the acetyl transferase p300. This modification was high in gene promoter regions that lack acetylation and associated with activation of genes expression (Zhang et al., 2019). As a large number of studies have demonstrated that lysine acylation possessed a diverse range of substrate proteins, however, no systematic analysis has been reported for lysine lactylation.

*Botrytis cinerea* is an worldwide economically important fungal pathogen with a wide variety of hosts (Williamson et al., 2007). Although the transcriptional regulation of many virulence genes in *B. cinerea* have been elucidated, the rare studies in protein level limited a deeper understanding of the molecular basis of *B. cinerea* pathogenesis. In this study, we conducted the first proteome-wide Kla analysis in *B. cinerea.* The screening identified 273 Kla sites from 166 proteins. The lactylated proteins were distributed in multiple compartments and associated with diversified biological processes, which do not have obvious roles in DNA-templated process. Besides, we identified 82 lactylation sites in 43 ribosomal proteins, indicating that Kla is an abundant PTM in the ribosome. Moreover, 12 lactylated proteins were found to be involved in diverse fungal pathogenetic pathways. Integration of these datasets offers a rich source for further characterizing the involvement of Kla in diverse cellular processes and pathogenesis of *B. cinerea*.

## Materials and Methods

### Protein Extraction From *B. cinerea*

The *B. cinerea* strain B05.10 conidia was collected and transferred into YEPD medium (2% peptone, 2% glucose, and 1% yeast extract) with shaking at 150 rpm for 12 h. The protein extraction was performed as previously described (Zhou et al., 2016). Protein extracted from four independent biological replicates was mixed together. Briefly, the harvested mycelia were ground into powder in liquid nitrogen. Then the powder sample was suspended by lysis buffer (0.1% protease inhibitor cocktail, 8 M urea, 3 μM Trichostatin A, 50 mM nicotinamide, 1% Triton-100, 2 mM EDTA, and 65 mM dithiothreitol) and then sonicated. After centrifugation at 15,000 × *g* in 4°C for 15 min, proteins were precipitated using15% cold TCA at −20°C for 2 h. Finally, after washed three times with cold acetone, the protein pellets from four independent biological experiments were combined and re-dissolved in urea buffer (8 M urea, 100 mM triethylammonium bicarbonate, pH 8.0). Then 2-D Quant kit (GE Healthcare) was used to determine protein concentration according to the manufacturer’s instructions.

### Western Blot Assay

The mycelia of the tested strains were grown in YEPD at 25°C for 12 h in a shaker treated with 0, 2, and 10 mM sodium lactate, respectively. The proteins were extracted as mentioned above. Western blot was performed as previously described (Xu et al., 2019). Briefly, proteins were separated by 12% SDS-PAGE and then transferred to PVDF membranes. After blocking with 5% milk, immunoblotting was conducted using pan anti-Kla multiclonal antibody (WM101, Micrometer Biotech Company, Hangzhou, China).

### Affinity Enrichment of Lysine Lactylated Peptides

For affinity enrichment, the *B. cinerea* proteins were firstly digested into peptides by trypsin (Zhou et al., 2016). Briefly, the trypsin was added at 1:50 trypsin-to-protein mass ratio at the first time overnight and added again at 1:100 trypsin-to-protein mass ratio for a further 4 h. The sample was separated into fractions by high PH reverse-phase HPLC using Agilent 300 Extend C18 column (5 μM particles, 4.6 mm ID, and 250 mm length) (Liu et al., 2016). The peptides were separated firstly into 80 fractions with a gradient of 2–60% acetonitrile in 10 mM (NH_4_)_2_CO_3_ (pH 10.0). Then, the peptides were combined into 8 fractions and dried by vacuum centrifuging. For Kla peptides enrichment, the tryptic peptides were dissolved in NETN buffer (1 mM EDTA, 0.5% NP-40,100 mM NaCl, and 50 mM Tris-HCl pH 8.0) and then separated into several fractions. Each fraction was incubated with pan anti-Kla antibody conjugated agarose beads overnight at 4°C with gentle shaking. After washing with NETN buffer and ddH2O, the bound peptides were eluted with 0.1% trifluoroacetic acid and then cleaned with C18 Zip Tips (Millipore).

### LC-MS/MS Analysis

The Kla peptides was dissolved and separated using a reversed-phase analytical column (Acclaim PepMap RSLC C18 column, Thermo Scientific). The gradient was composed of an increase from 2 to 10% solvent (0.1% formic acid in 98% acetonitrile) for 6 min, 10–20% for 45 min, 20% climbing to 80% in 7 min and then holding at 80% at least for 4 min, all at a flow rate of 250 nl/min on UPLC system. The peptides were subjected to ESI/NSI sources followed by MS/MS in Q Exactive^TM^ Plus (Thermo Scientific) coupled online to UPLC. The Orbitrap was used to detect whole peptides and ion fragments at a resolution of 70,000 and 17,500, respectively, with NCE set at 30. The electrospray voltage was set at 2.0 kV to analyze. Automatic gain control (AGC) was used to prevent overfilling of the ion trap. The m/z range was from 350 to 1,800 for MS scans. The MS fixed first mass was set at 100 m/z. The affinity enrichment and LC-MS/MS analysis were conducted in Micrometer Biotech Company (Hangzhou, China).

### Database Search

MaxQuant and Andromeda search engine (v.1.5.1.8) were used to analyze the raw data of MS/MS (Cox and Mann, 2008; Cox et al., 2009). The tandem mass spectra collected were searched against *B. cinerea* B05.10 database from UniProt. Mass errors of fragment ions and precursor were set as 0.02 Da and 10 ppm, respectively. Trypsin/P was specified as cleavage enzyme allowing up to 4 missing cleavage, 5 charges and 5 modifications per peptide. Carbamidomethylation on Cysteine was specified as fixed modification and lactylation on lysine was fixed as variable modification. The minimal peptide was set to seven, and the false discovery rate (FDR) threshold for modification sites and peptides were set as 1%. The Kla site localization probability of <0.75 was exclude.

### Bioinformatics Analysis

Gene ontology (GO) of lactylation proteome was performed from the UniProt-GOA database based on three categories: cellular component, molecular function, and biological process. The soft WoLF PSORT was used to predicate the subcellular localization of the lactylated protein (Horton et al., 2007). Protein secondary structures (β-strand, α-helix, coil) were analyzed by the online tool NetSurfP (Chou and Schwartz, 2011). Soft MoMo (motif-x algorithm) was used to analyze the sequences model of lactylated proteins composed of amino acids in distinct positions of modify-21-mers (10 amino acids up- and downstream of the Kla site) in all protein sequences. Kyoto Encyclopedia of Genes and Genomes (KEGG) database was employed to annotate protein pathway description (Kanehisa et al., 2004). Cytoscape software was used to analyze the protein–protein interactions which was obtained from the STRING database (Shannon et al., 2003; Szklarczyk et al., 2015). A two-tailed Fisher’s exact test was used to verify the enrichment of lysine lactylated proteins against all database proteins. All projects with a corrected *p*-valve < 0.05 is considered significant.

## Results

### Identification and Analysis of Lysine-Lactylated Sites and Proteins in *B. cinerea*

**FIGURE 1:**
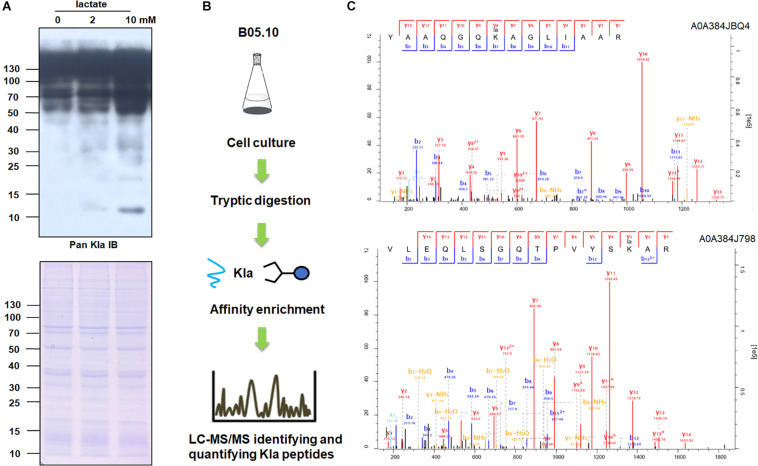
Overview of the experimental procedures of the Kla peptides in *Botrytis cinerea*. **(A)** Immunoblot analysis of lactylated proteins with pan anti-Kla antibody in *B. cinerea* treated with indicated doses of sodium lactate. The loading control by Coomassie blue staining was used to ensure that equal amounts of protein were loaded in each lane. **(B)** Systematic analysis of Kla in *B. cinerea*. **(C)** MS/MS spectra of two lactylated proteins, A0A384JBQ4 (60S ribosomal protein L11) and A0A384J798 (60S ribosomal protein L2/L8).

Kla is a previously unknown histone modification which was newly discovered in 2019 (Zhang et al., 2019). Kla dynamics in M1 macrophages are associated with gene expression and homeostatic response; however, nothing is known about the effects of Kla on non-histone proteins. To confirm the modification further, we carried out immunoblotting of lactylated proteins in *B. cinerea* using pan anti-Kla antibody (Figure 1A). The results showed that multiple protein bands spanning a wide mass range were detected, indicating the presence of non-histone Kla. Furthermore, as lactyl-CoA is the substrate for the Kla reaction, to investigate whether the lactylation level is affected by lactyl-CoA concentration *in vivo*, *B. cinerea* was cultured in YEPD liquid medium and treated with different concentrations of sodium lactate, which could be converted to lactyl-CoA. As shown in Figure 1A, the whole protein Kla levels were increased in a concentration-dependent manner in response to exogenous sodium lactate, which is in consistent with the dose-dependent increasing fashion of histone Kla in human (Zhang et al., 2019).

To extensively elucidate protein lactylation, a proteome-wide analysis of Kla was carried out in *B. cinerea*. Proteins extracted from mycelium were digested by trypsin and anti-lactyl lysine antibody was used to enrich the lactylated peptides, which were determined by following LC-MS/MS analysis. An overview of the experimental procedures was illustrated in Figure 1B. To confirm the dependability of the MS data, mass errors of the identified peptides were checked first. As shown in Supplementary Figure 1A, the distribution of mass errors was near zero, indicating the mass accuracy met the requirement for further analysis. Afterward, the length of most lactylated peptides were between 7 and 20, as expected of tryptic peptides (Supplementary Figure 1B). The above results suggested that the sample preparation met the standard.

A total of 273 Kla sites from 166 lactylated proteins were identified (Supplementary Table 1). The distribution of molecular weight of the identified proteins was first analyzed and the result demonstrated that 50% of them (80/168) were higher than 40 kd (Supplementary Figure 1C). Besides, MS/MS spectra of two lactylated peptides were shown in Figure 1C, representing ribosomal proteins, A0A384JBQ4 (60S ribosomal protein L11) and A0A384J798 (60S ribosomal protein L2/L8). The distribution of lactylated sites per protein was then calculated. As shown in Supplementary Figure 2, 103 (62%) proteins contained only one lactylation site, whereas 63 (38%) proteins had multiple lactylation sites, among which the percentage of proteins with two, three, four modification sites were 36 (21%), 17 (10%), and 7 (4%), respectively.

### Pattern Analysis of Lactylated Sites

**FIGURE 2:**
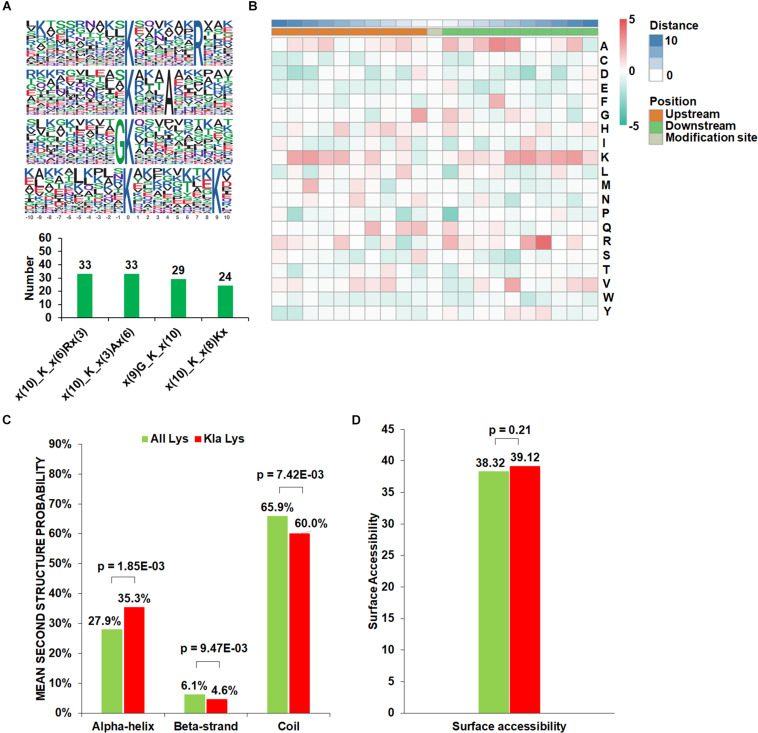
Properties of the Kla peptides in *B. cinerea*. **(A)** Lactylation sequence motifs for ±10 amino acids surrounding the Kla sites. Lactylation motifs were constructed with Motif-X software. The central K (at position 0) indicates the lactylation lysine. All the surrounding amino acid residues are indicated with the letters in different heights which is consistent with their frequencies in respective positions. Numbers of each conserved motifs were shown below. **(B)** Heatmap of the amino acid compositions of the Kla sites demonstrating the frequency of certain amino acids around the modified lysine. Red indicates high frequency and green means low frequency. **(C)** Probabilities of Kla in the structures of beta-strand, alpha-helix, and coil. **(D)** Predicted surface accessibility of Kla sites.

The context of amino acids flanking the Kla sites from −10 to +10 was assessed. A total of four conserved amino acid sequences were extracted, namely Kla_xxxxxxR (33 peptides), Kla_xxxA (33 peptides), GKla (29 peptides), and Kla_xxxxxxxxK (24 peptides) (Figure 2A). Afterward, we compared these conserved motifs of Kla with crotonylation and 2-hydroxyisobutyrylation in *B. cinerea*. Among them, GKla and Kla_xxxxxxxxK have been identified as crotonylation motifs, while Kla_xxxxxxR and Kla_xxxA was firstly found in *B. cinerea*, which may represent a characteristic of Kla in *B. cinerea.* To further analyze these motifs, heatmaps of the amino acid sequences around the lactylation sites were generated. The results showed that certain amino acid residues surrounding the Kla was markedly enriched. K residues was observed to be enriched in the −9 to −6 and +5 to +9 positions, while residues A, G, R was significantly enriched in +3 to +5, −1 to +1, and +6 to +7 positions, respectively (Figure 2B).

To investigate the relationship between lactylation and the presence of protein structures in *B. cinerea*, a structure analysis of the lactylated proteins was performed. As shown in Figure 2C, 40% of the lactylated sites were located in regions with ordered secondary structures. Among them, 35.3% were located in alpha-helix, and 4.6% were in a beta-strands. The residual 60% of the lactylated sites were distributed in disordered protein regions. Considering the distribution patterns of all lysine residues, we concluded that Kla may prefer alpha-helix structure than beta-strand or disordered regions in *B. cinerea*. In addition, the surface accessibility of Kla sites was further evaluated. The results showed that the exposure of lactylation sites on the protein surface is close to that of all lysine residues (Figure 2D). Therefore, Kla may not affect the surface properties of proteins in *B. cinerea*.

### Cellular Localization and Functional Enrichment Analysis of Lactylated Proteins

**FIGURE 3:**
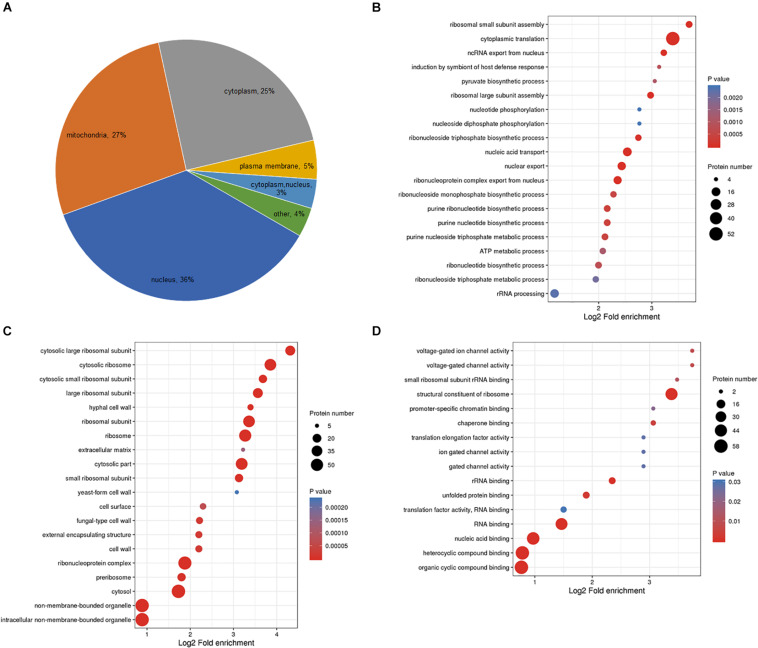
Classification of lactylated proteins based on subcellular localization analysis **(A)** and GO-based enrichment analysis of lactylated proteins according to biological process **(B)**, molecular function **(C)**, cellular component **(D)**.

To better characterize the lactylated proteins in *B. cinerea*, subcellular localization analysis of the lactylated proteins was conducted (Figure 3A and Supplementary Table 2). Most of the lactylated proteins were distributed in the nucleus (36%), mitochondria (27%), and cytoplasm (25%), demonstrating that the lactylated proteins were with diversified cellular distribution. Furthermore, we preformed functional enrichment analyses of GO (Gene Ontology). The biological process enrichment analysis demonstrated that the majority of lactylated proteins were involved in cytoplasmic translation (Figure 3B and Supplementary Table 3). Based on enrichment analysis of cellular component, proteins located to cytosolic ribosome were more likely to have lactylation (Figure 3C). In support of these observations, a large number of the lactylated proteins were related to structural constituent of ribosome in enrichment analysis of molecular function (Figure 3D). Further enrichment analyses of KEGG pathway obtained similar results, showing that the proteins associated with ribosome were more likely to be lactylated (Supplementary Table 4).

According to the above results, Kla in *B. cinerea* is abundant in ribosomal proteins. A summary of lactylated ribosomal proteins was presented in Table 1. We identified 82 Kla sites from 43 proteins, accounting for 26% of the total lactylated proteins in this study, suggesting important roles of lactylation in ribosomal functions. Taking together, our data indicate that lysine lactylated proteins are widely distributed and associated with various biological processes.

**TABLE 1:** List of lactylated ribosomal proteins in *B. cinerea.*

Protein accession	Protein description	Kla position
A0A384JCX3	40S ribosomal protein S3A	109
A0A384J453	40S ribosomal protein S2/30S ribosomal protein S5	49, 128, 194
A0A384J4J0	Ribosomal protein S4	8, 49, 60, 118, 129
A0A384J759	60S ribosomal protein L36	17
A0A384J798	60S ribosomal protein L11	52
A0A384J9H1	Ribosomal protein RPL1/RPL2/RL4L4	56, 220, 351
A0A384J9L6	Ubiquitin/40S ribosomal protein S27a fusion	6, 48, 63, 99
A0A384J9U0	60s ribosomal protein L15/L27	55
A0A384JBD4	Ribosomal protein S7	200
A0A384JBE3	60S ribosomal protein L10A	130, 133
A0A384JBQ4	60s ribosomal protein L2/L8	24, 145, 181, 221, 234
A0A384JD22	40S ribosomal protein S16	12
A0A384JD57	40S ribosomal protein S14	85
A0A384JD74	60s ribosomal protein L23	23
A0A384JD98	60S ribosomal protein L7A	18, 100, 252
A0A384JEF1	60s ribosomal protein L19	8, 19, 117
A0A384JFZ5	40S ribosomal protein S23	50, 56
A0A384JGS5	60S ribosomal protein L18A	158, 168
A0A384JI26	60S ribosomal protein L31	31
A0A384JJ19	40S ribosomal protein S11	41, 110
A0A384JJN2	40s ribosomal protein S27	22
A0A384JNZ0	40S ribosomal protein S29	27, 54
A0A384JSB5	60S ribosomal protein L44	32, 80
A0A384JSF0	60S ribosomal protein L14	106
A0A384JTU5	60s ribosomal protein L10	101
A0A384JU32	60s ribosomal protein L24	137
A0A384JU36	60S ribosomal protein L13a	54, 150
A0A384JUY8	60S ribosomal protein L22	46, 55, 74
A0A384JV25	40S ribosomal protein S7	119
A0A384JVB5	40S ribosomal protein S3	12, 80
A0A384JVY6	Ribosomal protein S18	25
A0A384JXL0	60s ribosomal protein L6	126
A0A384JY93	40S ribosomal protein S17	32, 59
A0A384JY93	40S ribosomal protein S17	32
A0A384JYI9	60S ribosomal protein L9	49
A0A384K0T1	60S ribosomal protein L37	13
A0A384K179	60S ribosomal protein L26	77, 89
A0A384K2W2	60S ribosomal protein L3 and related proteins	358, 365, 377, 385
A0A384K3A6	60S ribosomal protein L5	8, 165
A0A384K3C6	60s ribosomal protein L15	56
A0A384K3N7	60S ribosomal protein L22	22, 101
A0A384K438	40s ribosomal protein s10	9
A0A384K801	60S ribosomal protein L28	23, 46, 100, 129

*The protein accession numbers are from Uniprot database.*

### Analysis of Lactylated Proteins Related to Fungal Pathogenicity

**FIGURE 4:**
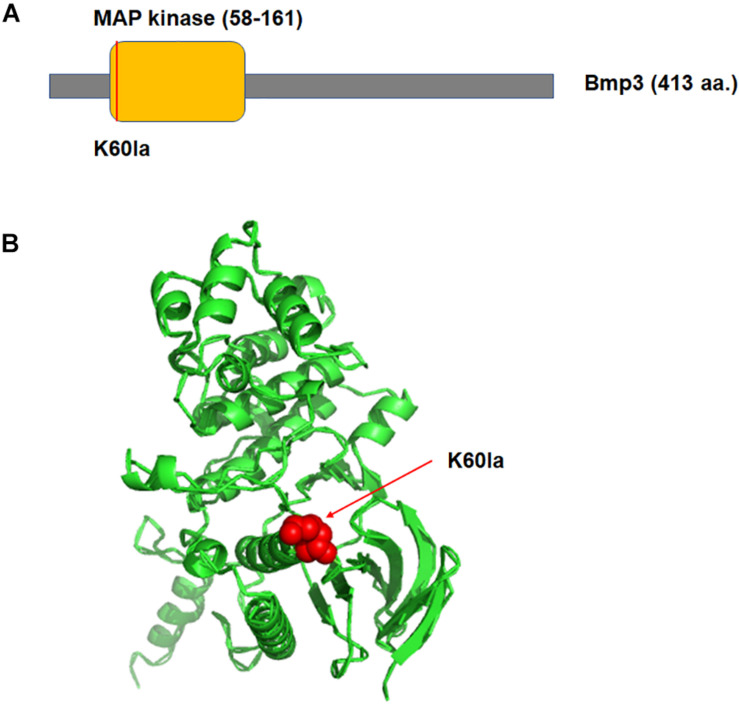
Illustration of functional domain **(A)** and three-dimensional structure **(B)** of Bmp3 with identified lactylation site. The structure was derived from PDB database. The lactylated lysine residues were indicated by red color.

Of the lactylated proteins identified in this study, some were reported to be involved in fungal virulence (Table 2). These proteins affected multiple aspects during the fungal infectious and pathogenetic process, including host adherence, signal transduction, primary nutrients transduction, molecular chaperons function, and ribosomal translation. For example, Bmp3, a mitogen-activated protein kinase involved in *B. cinerea* pathogenicity, was lactylated at K60 site. Importantly, the lactylated site was located in the functional domain, MAP kinase domain (Figure 4A). Further three-dimensional structure analysis demonstrated K60lac is in the center of the surrounding second structures (Figure 4B), which may have effect on the adjacent protein domains, and thus affect its function involved in pathogenicity.

**TABLE 2:** List of lactylated proteins associated with fungal pathogenicity.

Protein accession	Protein description	Kla position
A0A384K0E4	Chitin synthase	1352
A0A384JS06	Mitogen-activated protein kinase, Bmp3	60
A0A384J8S2	Citrate synthase	401
A0A384K3Z3	F0F1-type ATP synthase, alpha subunit	232
A0A384JCX1	Molecular chaperones HSP70 superfamily	75, 86, 185, 244, 355, 506, 544, 561
A0A384JG51	Molecular chaperones HSP70 superfamily	246
A0A384K0D3	Molecular chaperones HSP70 superfamily	98, 170
A0A384JTW8	Molecular chaperone HSP90 family	178, 196, 328, 485, 548, 616
A0A384K1B0	HSP90 co-chaperone p23	81
A0A384J7C4	Multifunctional chaperone (14-3-3 family)	141
A0A384JLW5	Translation initiation factor 5A (eIF-5A)	43, 76, 77
A0A384JP30	Translation initiation factor 5A (eIF-5A)	17, 132

*The protein accession numbers are from Uniprot database.*

### PPI Network of Lactylated Proteins in *B. cinerea*

**FIGURE 5:**
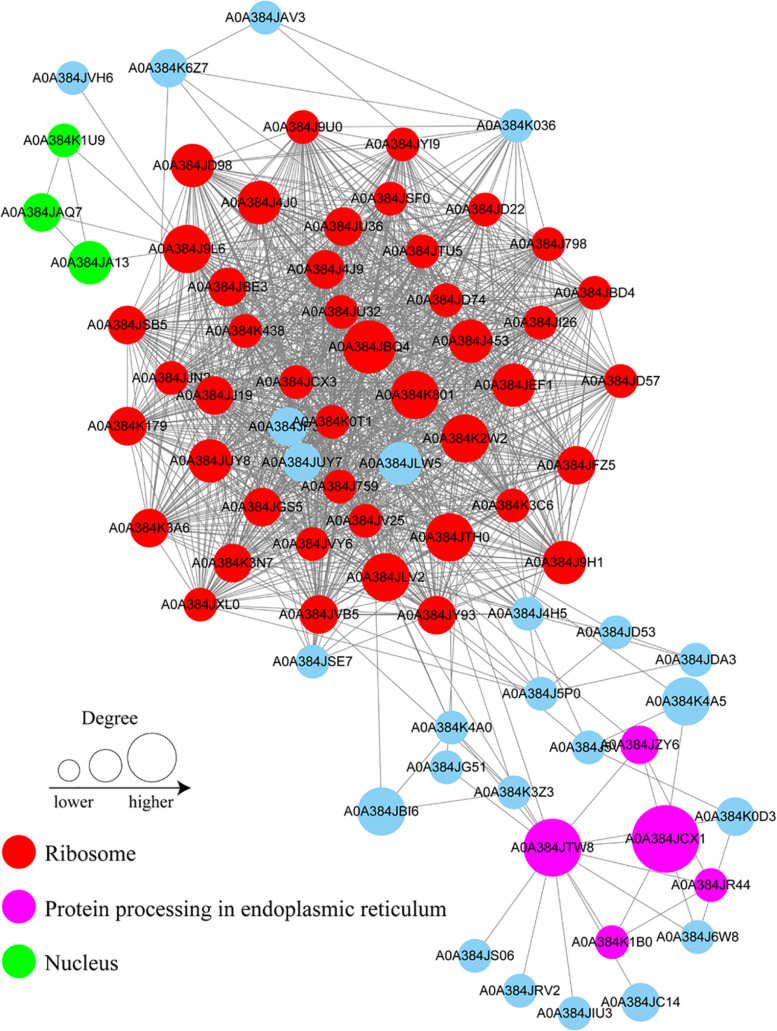
Protein interaction networks of the lactylation proteins in *B. cinerea*. Size of the node represent different number of modification sites. Different colors represent differential subcellular localizations: ribosomal protein (red); protein processing in ER (pink); nuclear protein (green).

To gain a better understanding of the cellular processes regulated by lactylation in *B. cinerea*, a PPI network of the identified lactylated proteins was assembled using Cytoscape software. A total of 76 lactylated proteins were mapped to the PPI network. These proteins were classified into three greatly interconnected clusters, including ribosome, protein processing in endoplasmic reticulum, and nucleus (Figure 5 and Supplementary Table 5). The most significantly enriched cluster was the ribosome, suggesting that ribosomal proteins are heavily modified by Kla. Overall, these findings indicate that physiological protein interactions among these complexes may contribute to their functional harmonization and cooperation in *B. cinerea*.

### Overlap of Lysine Lactylation, Crotonylation and 2-Hydroxyisobutyrylation in *B. cinerea*

**FIGURE 6:**
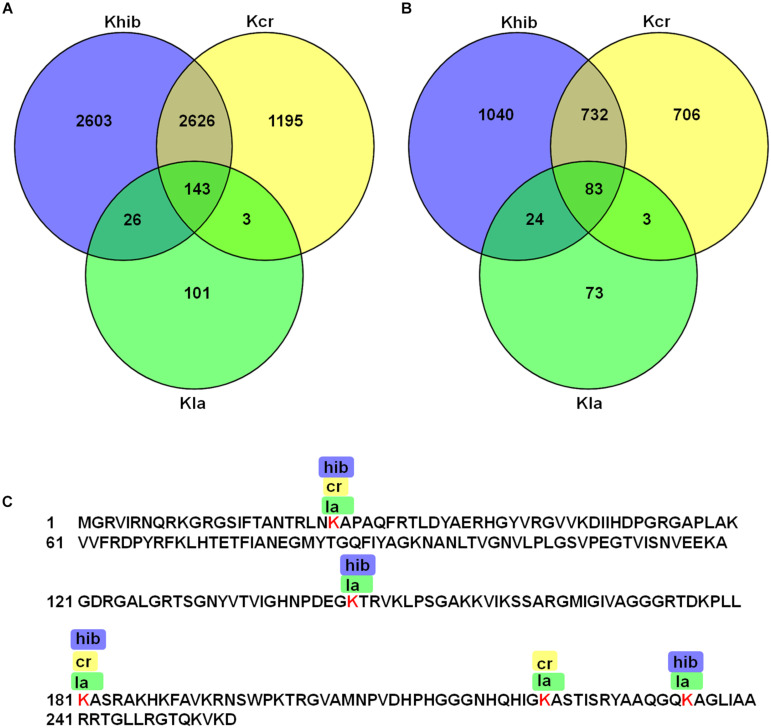
Overlap among Kla, Kcr, and Khib in *B. cinerea*. **(A)** Overlapped sites of Kla, Kcr, and Khib. **(B)** Overlapped proteins of Kla, Kcr, and Khib. **(C)** A representative protein showing the overlap of Kla, Kcr, and Khib sites.

In our previous research, 3967 lysine crotonylation (Kcr) sites on 1524 proteins and 5398 lysine 2-hydroxyisobutyrylation (Khib) sites on 1879 proteins were identified in *B. cinerea* (Supplementary Table 6). To determine whether Kla, Kcr, and Khib can occur on the same lysine residue, we compared the lysine lactylome with the crotonylome and 2-hydroxyisobutyrylome obtained previously in our laboratory. As shown in Figures 6A,B and Supplementary Table 6, 86 proteins were modified at the same 146 lysine site by lactylation and crotonylation. In addition, we found that 169 lactylation sites on 107 proteins were also 2-hydroxyisobutyrylated at the same position. Moreover, a total of 143 sites on 83 proteins were commonly modified by all three PTMs. A representative sample of overlapping among three PTMs was shown in Figure 6C. In the enzyme known as Ribosomal_L2_C domain-containing protein, 5 lactylation sites at K34, K145, K181, K221, and K234 were identified. Among these sites, K145 and K234 were also determined to be 2-hydroxyisobutyrylated; K221 was also crotonylated; K34 and K181 were modified by all three PTMs. These findings suggest that multiple PTMs can occur on the same lysine residue and may coordinately regulate the function of many proteins in *B. cinerea*.

## Discussion

Lysine lactylation was reported as a new type of PTM in 2019, involved in active gene expression in macrophages through regulating histone modification (Zhang et al., 2019). Non-histone Kla substrates have not been identified. Here we evaluated Kla modification of non-histone proteins in fungal pathogen *B. cinerea*. We identified 273 Kla sites in 166 proteins by immunoprecipitation with MS-based proteomics. This is the first large-scale dataset for lacylation of non-histone proteins and our study significantly expands the inventory of proteins containing this modification. The identification of previously unknown lysine-lactylated proteins from multiple cellular compartments, with diverse cellular functions including cyclic compound binding, structural constituent of ribosome, and hydrolase activity, implies that lysine lactylation is involved in the regulation of cellular pathways far beyond DNA-templated processes. Functional enrichment demonstrated that Kla is abundant in ribosomal proteins, indicating a potentially important link between lactylation and ribosomal functions. In addition, we identified 12 lactylated proteins associated with virulence of fungal pathogens, which may provide a new way to design drugs with high specificity and low toxicity to prevent and control gray mold disease.

Immunoblotting of lactylated proteins in *B. cinerea* showed that multiple protein bands were detected, most distributed higher than 40 kd (Figure 1A). However, the molecular weight distribution of lactylated proteins demonstrated that only around 50% of them (80/166) were higher than 40 kd (Supplementary Figure 1C). We thought there were two main reasons: (1) the efficiency of affinity enrichment is limited; (2) due to their short length, the majority of tryptic peptides are too small and thus generally not identified by MS, meaning that only a restricted segment of the proteome is covered (Tran et al., 2011; Tsiatsiani and Heck, 2015). Thus further researches are needed to focus on the use of a whole range of enzymatic and chemical cleavage methods, together with extensive fractionation, to maximize proteome sequence coverage.

In the pattern analysis of lactylated sites, we found that Kla may prefer alpha-helix structure than beta-strand or disordered regions in *B. cinerea*. Alpha-helix structure is the most abundant secondary structure in proteins and often located at surface of core, with hydrophobic residues on inner-facing side, hydrophilic on other side. It is reported that alpha-helix structure is involved in a variety of functions including DNA binding, protein interaction, and stability of membrane proteins (Mckay et al., 2018; Manav et al., 2019; Sang et al., 2019). Thus, the preference of Kla on alpha-helix suggested a potential role of Kla in the above mentioned biological pathways.

Histone PTMs have been shown to be important for many biological processes, including gene regulation, DNA repair, accurate genome organization, and DNA replication (Leach and Brown, 2012). Through mass spectrometry analyses, 26 and 16 histone Kla sites were identified from human and mouse, respectively (Zhang et al., 2019). In our study, the 273 Kla sites include 6 sites on histones, four of them conserved in human and two of them have not been reported thus far (Supplementary Figure 3). The total number of histones Kla sites were much less than human because (1) there are wide variations for the primary sequence of histone proteins between human and *B. cinerea*. (2) The Kla-antibodies used for enrichment may have been different, which could lead to varied recognition site. (3) The Kla may function distinctly on histones among different species.

Afterward, we identified 43 lactylated ribosomal proteins, accounting for 26% of the total lactylated proteins in this study. The Ribosome is a multi-component assembly responsible for translational control and are among the major sources for protein synthesis. Recent studies have suggested that PTMs of ribosomal proteins are important for biological functions. For example, ribosomal subunit L28 is modified by ubiquitination during S phase in yeast and is metabolically stable during translation with active ribosomal function (Spence et al., 2000). In addition, *N*-acetylation of ribosomal proteins is necessary to maintain protein synthesis function of ribosome in yeast (Kamita et al., 2011). A more recent study demonstrated that phosphorylation of ribosomal protein Rps15/Us19 is critical for the differential translation of specific mRNA in human (Simsek and Barna, 2017). Therefore, our data also indicate that Kla of ribosomal proteins is important for protein translational control and ribosome assembly.

Furthermore, 12 lactylated proteins were reported to be associated with fungal pathogenicity in our study. According to the cellular process these proteins were involved and their impact upon fungal virulence, we classified these proteins into five groups. First, during the infection process, the fungal cell wall plays an important role in the adherence to host tissues for colonization and host recognition. Chitin synthase (A0A384K0E4) which is essential for cell-wall chitin biosynthesis was found to be lactylated at site K1352. Disruption of the chitin synthase in filamentous fungi including *Colletotrichum graminicola*, *Wangiella dermatitidis*, and *Fusarium oxysporum* substantially impaired the fungal vegetable growth and pathogenicity (Amnuaykanjanasin and Epstein, 2003; Liu et al., 2004; Werner et al., 2007; Martín-Urdíroz et al., 2008). Second, Mitogen-activated protein kinases (MAPKs) play crucial roles in signal transduction during infection process and regulation of various aspects of pathogenic growth. MAPK (A0A384JS06) was identified lactylated at site K60. Knocking out this gene in *B. cinerea* caused severe defects in germination, vegetative growth, and pathogenicity (Rui and Hahn, 2007). Third, after establishment of colonization on the host, the fungi oxidized the primary nutrients from host cells through the tricarboxylic acid (TCA) cycle which is an essential metabolic network for ATP production. Citrate synthase is one of the rate-limiting enzyme of the TCA cycle. In our study, citrate synthase (A0A384J8S2) was found to be lactylated at site K401. A recent research demonstrated that citrate synthase contributes to infection and stress resistance of the stripe rust fungus (Li D. et al., 2018). A similar effect was detected with the F0F1-type ATP synthase (A0A384K3Z3, K232), which is required for carbon flexibility in *Candida albicans* to improve cell viability during the initial phase, thus contributes to fungal pathogenicity (Li S.X. et al., 2018). Fourth, molecular chaperones play important roles in protein folding, recognition, transport and degradation. Heat shock proteins (HSPs) and 14-3-3 proteins were relatively well-documented molecular chaperones thus far. Five HSP70/HSP90 proteins (A0A384JCX1, A0A384JG51, A0A384K0D3, A0A384JTW8, and A0A384K1B0) were lactylated at multiple lysine sites in this study. It is reported that HSP70s are essential for growth and pathogenicity of *Magnaporthe oryzae* (Yang et al., 2018), while HSP90 functions as a transcriptional regulator of conidiation and is required for virulence-associated stress responses in *Fusarium graminearum* (Bui et al., 2016). Recent study showed that the 14-3-3 proteins are involved in nitrogen response which affect virulence-associated behaviors in *F. graminearum* (Brauer et al., 2020). Finally, two translation initiation factor 5A (eIF-5A) proteins (A0A384JLW5 and A0A384JP30) were identified to be lactylated at multiple sites. Previous researches have found that hypusination of translation initiation factor-5A regulates *F. graminearum* virulence (Leach and Brown, 2012), indicating that Kla may affect the fungal virulence by regulating protein synthesis. Further efforts are required to define the mechanisms of lactylation on these virulence related proteins and the contribution to disease establishment and progression.

## Conclusion

We have conducted the first lactyl-proteome of *B. cinerea*, an important plant fungal pathogen. Our study reveals novel roles for Kla in the regulation of diverse cellular processes outside of the nucleus and provides a good resource for in-depth exploration of the functions of Kla in the fungal development and pathogenicity. Investigating the functions of the lactylation of the target proteins may help to design drugs with high specificity and low toxicity to prevent and control gray mold disease.

## Data Availability Statement

The mass spectrometry proteomics data have been deposited to the ProteomeXchange Consortium via the PRIDE partner repository with the dataset identifier PXD020746.

## Author Contributions

MG, NZ, and WL generated the hypothesis and planned the experiments. MG and NZ performed the experiments. NZ and WL wrote the manuscript. All authors contributed to the article and approved the submitted version.

## Conflict of Interest

The authors declare that the research was conducted in the absence of any commercial or financial relationships that could be construed as a potential conflict of interest.

## Supplementary Material

Supplementary Figure 1 **(A)** Mass error distribution of the Kla peptides. **(B)** Distribution of Kla peptides based on their length. **(C)** Molecular weight distribution of the identified proteins.

Supplementary Figure 2 Number of lactylation sites per protein in *B. cinerea*.

Supplementary Figure 3 Illustration of histones Kla sites identified in human and *B. cinerea*. Black triangles represent conserved sites, while red triangles represent unique sites in *B. cinerea.*

Supplementary Table 1 The identified Kla sites in *B. cinerea*.

Supplementary Table 2 Subcellular localization analysis.

Supplementary Table 3 GO enrichment based on molecular function, cellular component, and biological process.

Supplementary Table 4 Enrichment analysis of KEGG protein pathway.

Supplementary Table 5 Protein interaction network of the lactylated proteins.

Supplementary Table 6 Overlap among Kla, Kcr, and Khib in *B. cinerea*.
