# Supplementary figures and images for: Systematic Analysis of Lysine Lactylation in the Plant Fungal Pathogen Botrytis cinerea
Source: Front Microbiol. 2020 Oct 26;11:594743. doi: 10.3389/fmicb.2020.594743 (PMC7649125; doi:10.3389/fmicb.2020.594743)

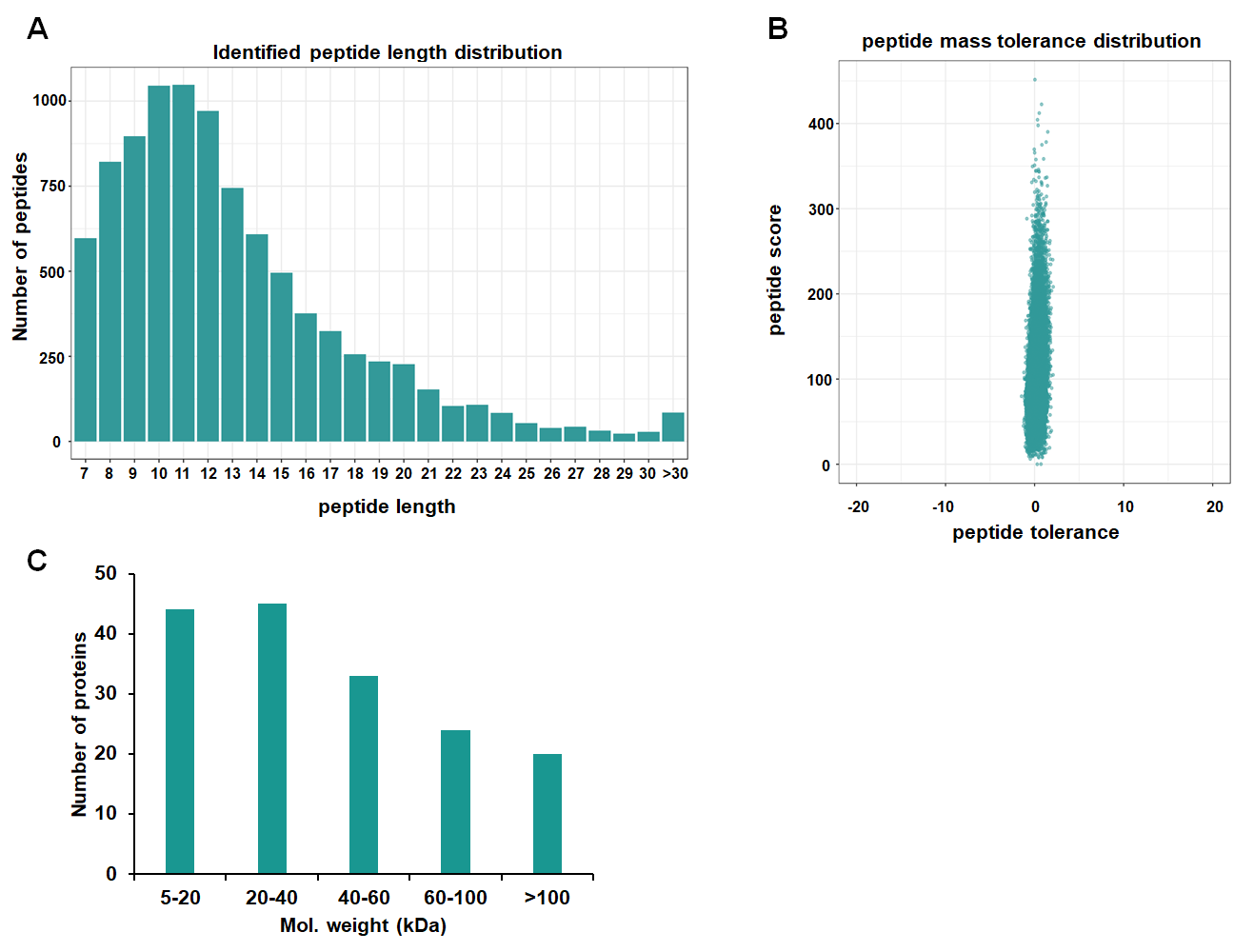

Supplement: Supplementary Figure 1 — (A) Mass error distribution of the Kla peptides. (B) Distribution of Kla peptides based on their length. (C) Molecular weight distribution of the identified proteins. [file Image_1.tif]

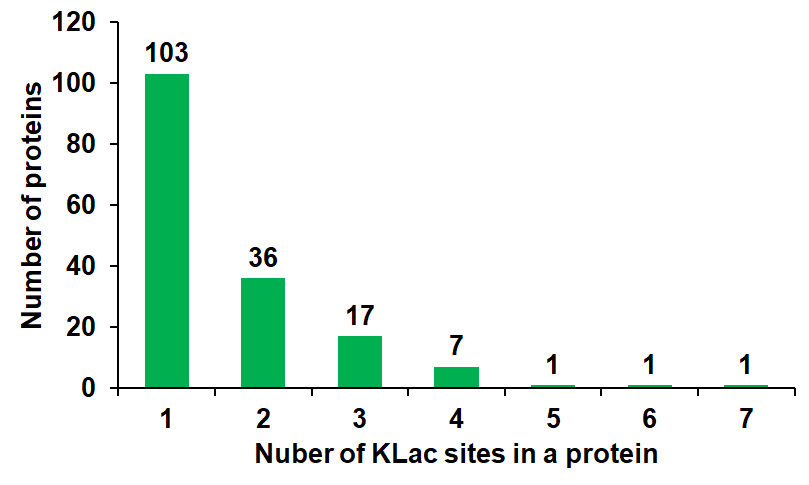

Supplement: Supplementary Figure 2 — Number of lactylation sites per protein in B. cinerea. [file Image_2.tif]

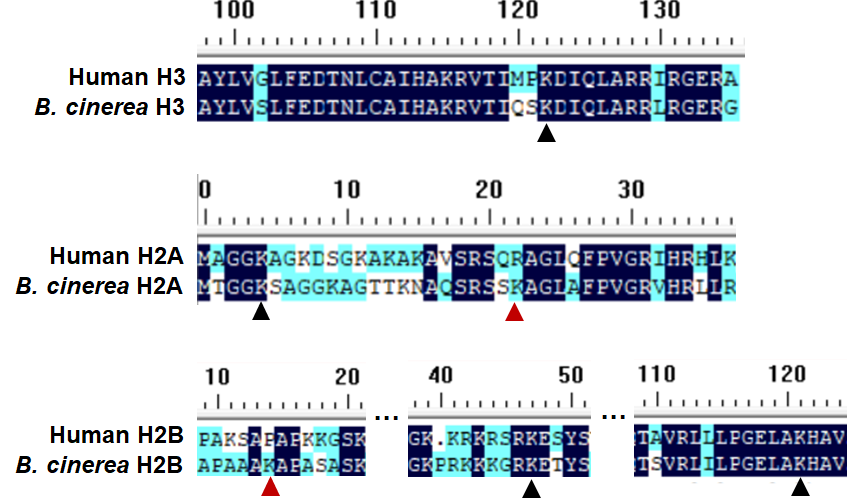

Supplement: Supplementary Figure 3 — Illustration of histones Kla sites identified in human and B. cinerea. Black triangles represent conserved sites, while red triangles represent unique sites in B. cinerea. [file Image_3.tif]
